# *Candida oesophagitis* as an Initial Manifestation of VEXAS Syndrome: Expanding Evidence for an Acquired Immunodeficiency Phenotype

**DOI:** 10.1002/jha2.70368

**Published:** 2026-07-25

**Authors:** Demi Rianne Vintges, Marina Weerheim, Faiz Karim

**Affiliations:** ^1^ Medical Doctor Internal Medicine Groene Hart Hospital Gouda the Netherlands; ^2^ Department of Internal Medicine Groene Hart Hospital Gouda the Netherlands

**Keywords:** *Candida oesophagitis*, opportunistic infections, VEXAS

1

To the Editor:

VEXAS syndrome is a recently described adult‐onset autoinflammatory disorder caused by somatic mutations in the *UBA1* gene, systemic inflammation, cytopenias, and bone marrow vacuolization. It is a prototypical hematoinflammatory disorder linking clonal haematopoiesis to severe, refractory inflammation. Opportunistic infections have not yet been sufficiently addressed [1]. We report a case that broadens the infectious spectrum and supports VEXAS as a potential acquired immunodeficiency.

We describe a 62‐year‐old man diagnosed with VEXAS syndrome based on recurrent fever, elevated CRP, relapsing polychondritis, cutaneous manifestations, MDS‐like bone marrow with cytoplasmic vacuolization, and a somatic UBA1 mutation, detected in peripheral blood with a variant allele frequency of 65%. This finding supports the diagnosis of VEXAS syndrome and suggests expansion of the mutant hematopoietic clone. Infectious (such as HIV, syphilis and tuberculosis) and autoimmune workup were negative. Endoscopy revealed biopsy‐confirmed *Candida oesophagitis* (Figure 1). Four weeks prior to the VEXAS diagnosis, a 2‐week course of fluconazole 200 mg was successfully completed. Initial treatment of VEXAS with prednisone was insufficient; subsequent weekly subcutaneous tocilizumab 162 mg led to good clinical response (Figure 2). Repeat endoscopy was not performed due to symptom resolution and subsequent loss to follow‐up after the patient returned to his home country.

**FIGURE 1:**
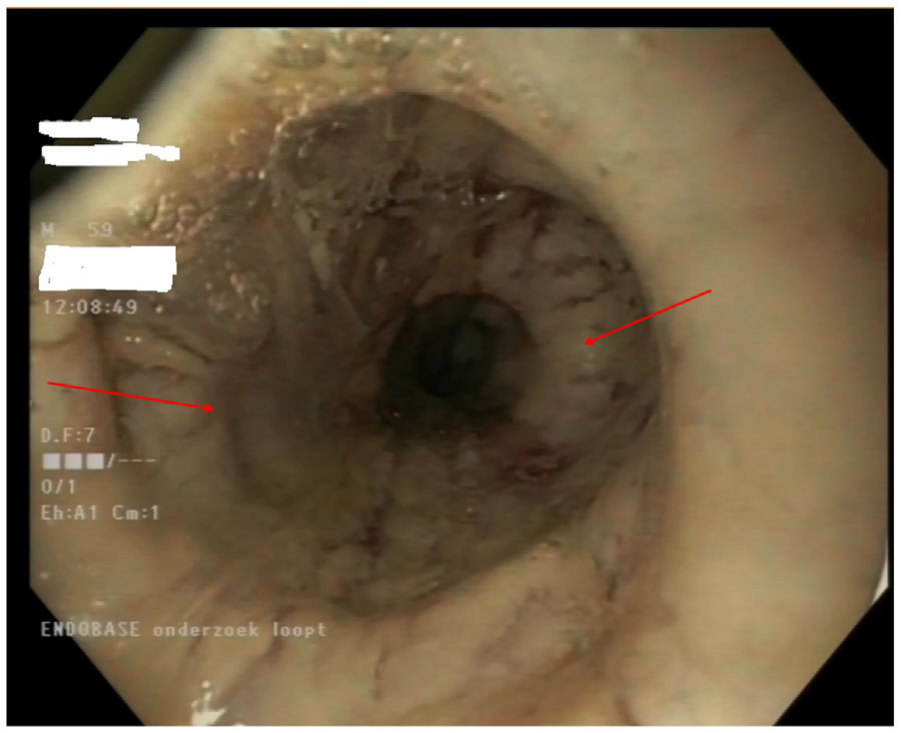
Endoscopic image of the oesophagus showing white plaques consistent with *Candida* infection (red arrows). Histological examination of the biopsy confirmed extensive *Candida* colonization. These findings illustrate the mucosal involvement in early VEXAS‐associated opportunistic infection in our case. Patient identifiers, including name and patient number, have been obscured with white to ensure privacy.

**FIGURE 2:**
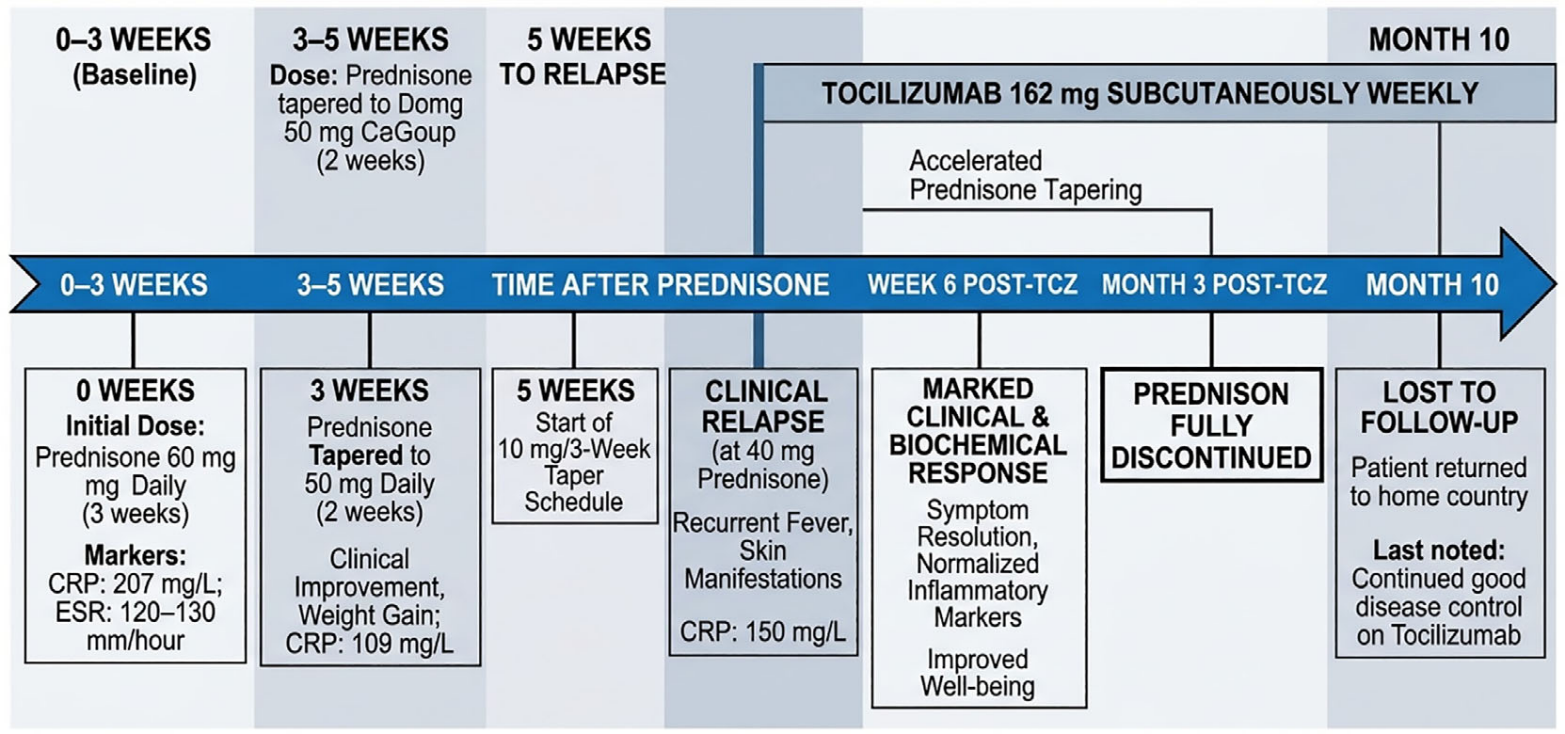
Timeline of clinical course and therapeutic response. Vertical arrows indicate time of key interventions, relapses and outcomes. Shaded area above the timeline shows concurrent treatment with tocilizumab. The patient was started on prednisone 60 mg daily, followed by tapering to 50 mg and subsequent gradual dose reduction. A clinical flare occurred at a prednisone dose of 40 mg, characterized by recurrence of fever, skin manifestation and rising inflammatory markers, after which subcutaneous tocilizumab (162 mg weekly) was initiated. This resulted in rapid clinical and biochemical remission within approximately 6 weeks, allowing complete discontinuation of prednisone. Tocilizumab was continued with sustained disease control until approximately 10 months, when the patient was lost to follow‐up. TCZ, tocilizumab.

VEXAS results from somatic *UBA1* mutations, affecting the ubiquitin‐activating enzyme E1, a key regulator of protein degradation, stress responses and immune signalling. This dysregulates innate immunity, myeloid function and drives systemic inflammation. Beyond autoinflammation, *UBA1*‐defects may impair host defence. Monocytes and macrophages, crucial for antifungal immunity via phagocytosis and cytokine signalling, may exhibit altered function in VEXAS, compromising mucosal antifungal responses and microbial homeostasis, including defence against *Candida*‐species [2, 3]. Opportunistic infections are reported in VEXAS, including Pneumocystis jirovecii, non‐tuberculosis mycobacteria and invasive fungi [4], often linked to prolonged immunosuppressive therapy. Our case is notable for *C. oesophagitis* at initial presentation, suggesting that intrinsic immune dysregulation in VEXAS may independently increase susceptibility to opportunistic infections. In addition to impaired cellular immune function, chronic systemic inflammation may contribute to disruption of mucosal‐barrier integrity. Persistent inflammatory cytokine activation may alter epithelial defence mechanisms, further increasing susceptibility to fungal colonization and invasion. The combination of innate immune dysfunction and mucosal barrier impairment provides a plausible mechanistic explanation for mucosal candidiasis as an early clinical manifestation of VEXAS [5].

Recognition of infections in VEXAS has important implications. Opportunistic infections in patients with symptoms consistent with VEXAS should prompt evaluation and genetic testing. Better definition of infection risk may help guide antimicrobial prophylaxis, especially during prolonged immunosuppression [5]. In the context of emerging therapeutic strategies, awareness of potential infectious complications is essential to ensure appropriate monitoring and preventive care. Further studies are required to clarify the incidence, spectrum and pathophysiological mechanisms of infections in VEXAS syndrome. To our knowledge, *Candida* infection has not previously been reported in association with VEXAS, although it may have been underestimated.

Additional case reports or studies on infectious manifestations may clarify whether mucosal candidiasis is an under‐recognized feature of VEXAS and support its overlap with autoinflammation and acquired immunodeficiency.

## Author Contributions

D.R.V. and F.K. initially determined the content of this correspondence. D.R.V. and M.W. developed the concept and drafted the correspondence in accordance with the suggestions of the British Journal of Haematology. F.K. prepared the figure, critically revised the manuscript and finalized the submitted version.

## Funding

The authors have nothing to report.

## Conflicts of Interest

The authors declare no conflicts of interest.

## Data Availability

Data is available upon request.
